# EHMTI-0159. Assessment of functional health and well-being in headache patients: the effect of individual-based physical therapy

**DOI:** 10.1186/1129-2377-15-S1-D29

**Published:** 2014-09-18

**Authors:** J Hirsvang, LS Kroell, N Caspersen, BK Madsen, RH Jensen

**Affiliations:** 1 Dept. Neurology Glostrup Hospital, Danish Headache Center, Copenhagen, Denmark

## Background

Patients with tension-type headache (TTH) and/or migraine are often referred to physical therapy. Only few studies have evaluated the efficacy of individual-based exercise program.

## Aim

To evaluate the effect of individual-based exercise on functional health and well-being, headache frequency and pain intensity in patients suffering from TTH and/or migraine.

## Methods

During an 18 months period 282 consecutive patients entered an open label uncontrolled study in a multidisciplinary headache centre. Diagnoses were according to ICHD-3 beta. Functional health and well-being were measured by using Headache Impact TestTM (HIT-6), scores ranged from 36 (no impact) to 78 (severe impact). Pain intensity was recorded on an 11-point numeric rating scale(NRS-11), 0 = no pain; 10 = worst pain imaginable.

## Results

So far, 40 women and 14 men, mean age 39.4, completed the study. 28 were diagnosed with TTH, 4 with migraine and 22 with both TTH and migraine.

In TTH-patients HIT-6 decreased from 59.6 to 54.4(p<0.01); frequency decreased from 24.9 to 18.0 days/month(p<0.01); pain intensity decreased from 5.8 to 3.9 NRS-11(p<0.01).

In patients with combined TTH and Migraine HIT- 6 decreased from 61.3 to 58.8(p=0.06); the TTH-frequency decreased from 20.6 to12.6 days/month(p<0.01), and their migraine frequency from 4.0 to 2.3 days/moth(p=0.03); whereas pain intensity was uneffected (TTH: p=0.09, Migraine: p=0.15).

## Conclusion

Individual-based exercise has shown a positive effect on functional health and well-being, headache frequency and pain intensity in severely affected patients especially in TTH and less for combined Migraine and TTH. Randomised controlled studies are needed to confirm these findings.

No conflict of interest.

